# Select physical performance measures and driving outcomes in older adults

**DOI:** 10.1186/s40621-017-0110-2

**Published:** 2017-05-08

**Authors:** Thelma J. Mielenz, Laura L. Durbin, Jodi A. Cisewski, Jack M. Guralnik, Guohua Li

**Affiliations:** 1 Department of Epidemiology, Columbia University Mailman School of Public Health, 722 West 168th St., New York, NY 10032 USA; 2 Center for Injury Epdemiology and Prevention, Columbia University Medical Center, New York, NY USA; 3 Division of Gerontology, Department of Epidemiology and Public Health, University of Maryland School of Medicine, Baltimore, MD USA; 4 Department of Anesthesiology, College of Physicians and Surgeons, Columbia University Medical Center, New York, NY USA

**Keywords:** Driving, Function, Health, Mobility, Transportation

## Abstract

**Background:**

Improving physical functioning may be a future intervention to keep older adults driving safely longer as it can help maintain both physical and cognitive health longer. This systematic review assesses the evidence on the association between three physical functioning measures: the Short Physical Performance Battery, the Timed Up-and-Go test, and the Rapid Pace Walk with driving outcomes in older adults.

**Methods:**

Older adult studies published between 1994 and 2015 that included the Short Physical Performance Battery, the Timed Up-and-Go test, or the Rapid Pace Walk as a measure of physical functioning and included a driving-related outcome were identified through a comprehensive search and reviewed following Preferred Reporting Items for Systematic Reviews and Meta-Analyses guidelines.

**Results:**

Thirteen studies involving 5,313 older adults met the inclusion criteria. Lower Short Physical Performance Battery scores were associated with reduced driving exposure and increased cessation in all three Short Physical Performance Battery studies. The Timed Up-and-Go test was not associated with the driving outcomes (cessation, ability, crashes, and citations) in either of the two Timed Up-and-Go studies. Poorer Rapid Pace Walk scores were associated with decreased driving ability in two studies and with reduced driving exposure in one study, but not associated with driving ability, crashes, citations, or cessation in the remaining five Rapid Pace Walk studies.

**Conclusions:**

The Timed Up-and-Go test measure appears not to be a useful measure of physical functioning for the driving outcomes included here. The Rapid Pace Walk may be useful in studies of driving ability and exposure. More driving studies should consider using the Short Physical Performance Battery to determine if it may be useful as a risk factor assessment for identifying individuals at risk of certain driving outcomes.

## Review

Driving a motor vehicle is an important component of the lives of most older adults in the United States, and this transportation activity allows older adults to maintain their independence and mobility. Even after adjusting for the influence of sociodemographic and health-related factors, driving cessation among older adults is strongly associated with decreased out-of-home activity levels (Marottoli et al. 2000). Quality of life is typically reduced for older adults when they cease driving and certain vulnerable populations, such as women and financially disadvantaged individuals, often suffer the most from giving up their driving mobility (Oxley and Whelan 2007).

Access to public transportation is still lacking especially in rural areas, thus older adults will continue to drive in order to keep their independence (Miller et al. 2016). Despite the benefits of continued driving, there are legitimate concerns regarding the ability of older drivers to continue driving safely.

Avenues are being explored for interventions to keep older adults driving safely as long as possible. Intervention studies are emerging exploring the efficacy between exercise training and driving outcomes (Marmeleira et al. 2011).

With the eventual progression to intervention studies, it is first important to understand the association between physical performance and certain driving outcomes. Second, does one physical performance measure, as there are many, have a stronger magnitude of effect on certain driving outcomes? Therefore, the objective of this review is to assess the evidence in the research literature on the association of three validated lower extremity strength and balance physical functioning measures and driving outcomes in older adults, including: driving exposure, cessation, crashes, citations, and ability. These three measures include one battery and two stand-alone measures: the Short Physical Performance Battery (SPPB), the Timed Up-and-Go (TUG) test, and the Rapid Pace Walk (RPW).

## Methods

This systematic literature review includes a narrative synthesis and adheres to reporting standards laid out in Preferred Reporting Items for Systematic Reviews and Meta-Analyses (or PRISMA) guidelines (Moher et al. 2009). It is registered with PROSPERO under the registration number CRD42015026613.

### Driving outcomes

The driving outcomes discussed below are those included in articles that met our eligibility criteria and acquired in full-text.

#### Driving exposure and cessation among older adults

Research suggests that there are serious consequences from reduced driving exposure and increased driving cessation among older adults. Fonda et al. (2001) note that older adults who reduce or cease driving are at a greater risk for worsening depressive symptoms, even when they have a spouse who is able to drive them instead. It is even possible that driving cessation increases an individual’s risk of entering long-term care (Freeman et al. 2006).

Older adults cease driving for a variety of reasons, which range from financial, vehicle access, and psychosocial reasons to various age-related medical concerns (Anstey et al. 2006; Carr et al. 2006; Dellinger et al. 2001; Edwards et al. 2008; Freeman et al. 2005; Marottoli et al. 1993; Sims et al. 2007). Medical concerns that impact driving decisions among older adults include: vision, Parkinson’s disease, stroke-related residual paralysis or weakness, syncope, diabetes, stroke, depression, neurologic disease, congestive heart failure, arthritis, and taking sedating medications (Campbell et al. 1993; Carr et al. 2006; Edwards et al. 2008; Freeman et al. 2005; Marottoli et al. 1993; Ragland et al. 2004). In addition to specific medical diagnoses, physical performance is a reliable health-related predictor of driving cessation (Ackerman et al. 2008; Edwards et al. 2008; Sims et al. 2007).

#### Crashes, citations, and ability among older adults

Crashing is a concerning driving outcome for older adults, as crash outcomes are more often deadly for this population than for younger adults (Lyman et al. 2002). Crashes are an outcome that can arise from a general lack of driving ability. However, less severe outcomes (including errors that result in citations and driver errors that may go unnoticed) can indicate limited driving ability that should be addressed before crashes occur. In addition to errors that are clearly citation-worthy, other errors include failing to check the rear-view mirror, driving while distracted, or failing to brake when appropriate (Emerson et al. 2012). Such actions result in near-crashes that go unreported and are difficult to quantify, with near-crashes being defined as circumstances requiring any vehicle, pedestrian, or other actor on the road to make an evasive maneuver in order to avoid crashing (Dingus et al. 2006).

Crashes and poor driving ability among older adults are frequently associated with medical or chronic health conditions, including: alcohol abuse and dependence, dementia, depression, schizophrenia, epilepsy, cardiovascular disease, diabetes mellitus, cerebrovascular disease, traumatic brain injury, musculoskeletal disorders, obstructive sleep apnea, vision disorders, and the use of certain medications (Marshall 2008). However, using these diagnoses alone to determine fitness-to-drive would overly restrict safe drivers, as these conditions are only slightly to moderately associated with an increased crash risk, and so other factors related to physical and cognitive health must be considered such as the presence of multiple medical conditions and varying levels of disease severity (Miller et al. 2016; Marshall 2008).

### Physical performance measures

Physical performance is operationally defined for this review as an objective performance measure of physical functioning. More specifically, these objective physical performance measures have individuals perform standardized tasks and performance on these tasks is evaluated according to predetermined criteria, which could include the timing of the activity or a counting of repetitions, depending on the type of task (Guralnik et al. 1989).

#### Short physical performance battery

The Short Physical Performance Battery (SPPB), created by Guralnik et al. (1994), is used to assess balance and physical functioning, specifically lower extremity function. Guralnik and colleagues adapted previously used measures with the aim that one trained lay interviewer with limited space and time (10–15 min) can conduct the SPPB, while ensuring the safety of the participants. There are three major components of the SPPB: standing balance (standing with feet together in three positions of increasing difficulty: side-by-side, semi-tandem, and tandem), walking speed (usual speed on a four meter course), and ability to rise from a chair (time to rise five times from a chair with arms folded across the chest). Scores of zero (inability to carry out task) to four (best performance possible) are assigned for each of the three tasks, and these are summed to create a final SPPB score (range zero to 12). Lower overall scores indicate poorer physical functioning.

Guralnik et al. (1994) validated the SPPB in a study of more than 5,000 older adults who were aged 71 years and older. Each test and an overall SPPB score were strongly associated with self-report of disability. Both self-reported disability and SPPB scores were predictors of short-term mortality and nursing home admission. Individuals self-reporting themselves as high functioning were able to be placed on a gradient of risk for mortality and nursing home admission by using their SPPB scores. Guralnik et al. (1995) further determined that SPPB scores can predict onset of disability within a nondisabled older adult population.

Since the development of the SPPB, both its reliability and sensitivity to change have been confirmed, and it is a widely used physical functioning measure in older adult research (Ostir et al. 2002). Multiple population studies of aging have utilized the SPPB, including the Established Populations for Epidemiologic Studies of the Elderly Study, National Health and Aging Trends Study, and the Lifestyle Interventions and Independence for Elders Study Randomized Clinical Trial (Guralnik et al. 1994; Kasper et al. 2012; Pahor et al. 2014). Studies also confirmed a high validity for the SPPB as a measure of functional status and reported that the SPPB can predict hospitalizations and length of hospital stay, identify patients who are at a higher risk of poor outcomes after being discharged from a hospitalization, and predict declines in function and health status (Penninx et al. 2000; Studenski et al. 2003; Volpato et al. 2008; Volpato et al. 2011). The SPPB is further a known predictor for mortality (Rolland et al. 2006; Cesari et al. 2008; Ostir et al. 2007).

#### Timed up-and-go test

The Timed Up-and-Go (TUG) test is a timed derivative of the Get-Up-and-Go test, which was created by Mathias et al. (1986). In the TUG, participants are observed and timed as they rise from an arm chair, walk 3 m, turn, walk back, and sit back down (Podsiadlo and Richardson 1991). Podsiadlo and Richardson (1991) found that this timed measure was a risk marker for an older adult’s ability to go safely outside alone. The measure has content validation since it focuses on physical actions that are used in daily life and concurrent validation, as it correlates with other established measures of balance and functional ability including measures on the Berg Balance Scale, gait speed, and measures from the Barthel Index of Daily Living Scale (Bennie et al. 2003; Freter and Fruchter 2000; Podsiadlo and Richardson 1991).

Since its creation, inter-rater and test-retest reliability of the TUG is confirmed (Shumway-Cook et al. 2000; Noren et al. 2001). It is one of the two measures recognized in the *Clinician’s Guide to Assessing and Counseling Older Drivers*, which was released by the National Highway Traffic Safety Administration and the American Geriatrics Society (2016). The TUG is used most often in falls-related research, and TUG scores are found to be both sensitive and specific for identifying older adults prone to falling (Shumway-Cook et al. 2000). The TUG can be completed by most older adults and is a quick and easy-to-administer test, so it is used frequently with older adult populations. It is included in the US Centers for Disease Control and Prevention tool kit for clinical screening of fall risk called Stopping Elderly Accidents, Deaths, and Injuries (or STEADI) and is also part of the fall-focused physical examination for the Annual Medicare Wellness Visit (Phelan et al. 2015).

#### Rapid pace walk

The Rapid Pace Walk (RPW) is a rapid version of the Usual Pace Walk, first appearing in the literature in an older adult driving-related study (Marottoli et al. 1994). Participants are asked to walk 10 ft away and back at the fastest pace at which the participants feel safe and comfortable (Marottoli et al. 1994). Since its first use, the RPW is frequently used in driving studies and is the other measure of note along with the TUG in the *Clinician’s Guide to Assessing and Counseling Older Drivers*, which was released by the National Highway Traffic Safety Administration and the American Geriatrics Society (2016). The RPW is included in the Assessment of Driving-Related Skills (ADReS), which is a test battery that consists of vision, cognition, and motor/somatosensory function measures that assess skills necessary for safe driving (Carr et al. 2010). A study on the stability of physical assessment measures found that the RPW had a moderate relative reliability and low coefficients of variability (CV) values (Smith et al. 2013).

### Eligibility

Studies were eligible for inclusion in this systematic review if they: 1) included adults aged 50 years and older; 2) included at least one driving-related outcome; 3) used the full SPPB, the TUG test, or the RPW, or a modified version of one of these measures, as an objective tool to measure physical functioning, and examined analytically a possible connection between the SPPB, TUG, or RPW and a driving outcome; 4) were published in the English language; 5) were published between the years 1994 and 2015, inclusive; and 6) used an epidemiological design (cross-sectional, cohort, or case–control). Acceptable studies were analytical in nature, and so all qualitative studies, patents, letters, commentaries, reviews, editorials, and opinion pieces were excluded.

### Search strategy, data sources and extraction

A research librarian was consulted for constructing the search strategy and terms. All retained articles were pulled from the following electronic databases through a comprehensive search on November 11, 2015: PsycINFO®, EBSCO, CINAHL, Medline OVID, PubMed, Scopus, and TRID. One author (LD) screened all article titles and abstracts using the inclusion and exclusion criteria previously stated. Studies with unclear eligibility were reviewed in full-text using these criteria.

The MeSH (Medical Subject Heading) term “automobile driving” was used in conjunction with “Short Physical Performance Battery,” “Timed Up and Go,” and “Rapid Pace Walk,” as well as the abbreviated versions “SPPB,” “TUG,” and “RPW.” After examining the returned articles, the non-MeSH term “driving” was also used in conjunction with all of the preceding terms and abbreviations to determine if any articles had been previously overlooked. In order to obtain any articles that were not captured with the specific physical performance terms, the term “geriatric assessment” was then used in conjunction with “automobile driving” and with “driving.” For most databases, the “all text” or “all fields” option was selected. For the Scopus database, due to its diverse scientific content, the “article title, abstract, keywords” option was selected.

### Quality assessment

The quality of included studies was evaluated using the Newcastle-Ottawa Quality Assessment Scale (Wells et al. 1999). The scale is only directly applicable to case-control and cohort studies, so for cross-sectional studies the reviewers modified the scale to exclude consideration of the follow-up period and absence of outcome at the beginning of the study (Chihuri et al. 2015; Herzog et al. 2013). The best score possible depended on study design, with lower scores indicating poorer study quality. Ten was the best possible score for cross-sectional studies, whereas nine was considered the best score for cohort studies.

## Results

**Fig. 1:**
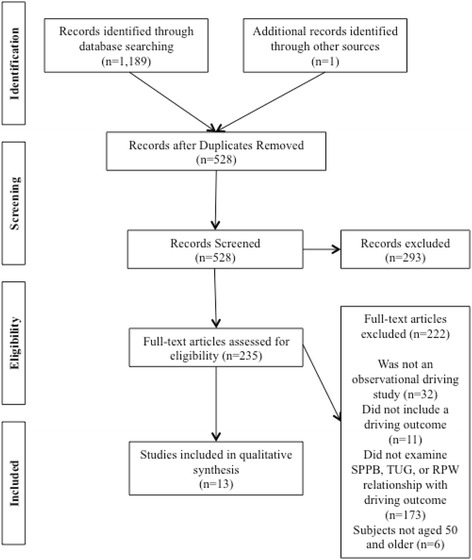
Flow diagram of the study selection included in the systematic review of SPPB, TUG, and RPW predicting older adult driving outcomes

Across the six databases, 1,189 results were returned. One additional resource was identified, a 1994 study by Marottoli et al., which is the first study where the RPW measure is mentioned (Marottoli et al. 1994). A total of 662 results were removed for being duplicates, leaving 528 citations to be screened. Studies were then excluded that clearly did not meet eligibility criteria and 235 articles were assessed for full-text eligibility. Thirteen studies from the remaining citations met the eligibility criteria and were retained to be included in the systematic review (Fig. 1). No studies were excluded for reporting negative findings.

### Study characteristics

Ten studies were conducted in the United States (Alabama, Connecticut, Florida, Iowa, Maryland, and Missouri), one in the United Kingdom (Bristol), and two in Canada (British Columbia), Australia (Queensland), and New Zealand (Wellington) (Table 1). Two publications reported outcomes from the same sample in Iowa City, Iowa and two publications reported outcomes from the same Maryland Older Drivers Project (Ball et al. 2006; Dawson et al. 2010; Edwards et al. 2010; Emerson et al. 2012). All 13 studies included both men and women and the participants in all studies were at least 52 years old. Ten of these studies only included participants that were at least 65 years old. Participant recruitment for each study is reported in Table 1. The study types varied, including five cross-sectional studies and eight cohort studies. One of the cohort studies included only baseline data, which were analyzed cross-sectionally (Langford et al. 2013). Only one study included a modified physical performance measure (Gill et al. 2012). The studies in this review had various socio-demographic covariates in their analyses, which included age, sex, education, and race (Table 2).

**Table 1:** Characteristics of driving studies using the SPPB, the TUG, or the RPW as a measure of physical functioning

First author, Year	Study participants	Data source	Study design	Location	Study time period	Type of driving outcome	Source of driving outcome information
SPPB							
Davis, 2011	214 participants aged 70 years and older	Project OPAL (Older People and Active Living)	Cohort study	Bristol, UK	2007–2008	Driving exposure (number of car trips as a driver per week)	Combination of accelerometry (Actigraph GT1Ms) and daily trips logs
Gill, 2012	507 community-dwelling adults aged 70 years and older who were active drivers or nondisabled in walking a quarter mile	Precipitating Events Project	Cohort study	Greater New Haven, Connecticut	1998–2009	Driving cessation (long-term disability in driving a car, indicated by not driving in the past 6 months)	Participant responses during monthly interviews
Sims, 2007	649 community-dwelling adults aged 65 years and older who reported driving at baseline	University of Alabama at Birmingham (UAB) Study on Aging (SOA)	Cohort study	Five central Alabama counties	1999–2003	Driving cessation	Participant responses during 2-year telephone follow-up interview
TUG							
Dawson, 2010	111 participants aged 65 and older who were current drivers	Participants recruited through announcements throughout the community	Cross-sectional study	Iowa City, Iowa	Not specified	Driving ability (safety errors per drive)	Video review of performance on a 35-mile road test in an instrumented vehicle
Emerson, 2012	100 participants aged 65 and older who were current drivers	Participants recruited through announcements throughout the community	Cohort study	Iowa City, Iowa	Not specified	Driving cessation, citations, and crashes (time to driving event over a length of follow-up ranging from 3 to 8 years)	Cessation was determined by participant or family report at end of follow-up period (or, if needed, by driving records, ARGOS drive status, death date, or the Driving Habits Questionnaire (DHQ)); citations were tracked with yearly requests to Iowa DOT driving records; crashes were determined from DHQs at annual visits and from Iowa DOT driving records
RPW							
Ball, 2006	1,910 participants aged 55 years and older who were current drivers	Maryland Older Drivers Project	Cohort study	Maryland	1998–2003	Driving crashes (at-fault motor vehicle collision involvement during follow-up period of between 4.18 and 5.13 years)	Maryland MVA Administration of Driver Safety Research Office crash records
Classen, 2013	195 community dwelling current older drivers aged 65 years and older	National Older Driver Research and Training Center (NODRTC) study and	Cross-sectional study	North-central Florida	2004–2006 and 2010–2012	Driving ability (passing or failing an on-road driving test)	Road test administered by a certified driving rehabilitation specialist (CDRS)
Carr, 2011	99 participants aged 52 years and older with dementia who were current drivers	Participants recruited through physician referral	Cross-sectional study	St. Louis, Missouri	2007–2009	Driving ability (passing or failing the Washington University Road Test)	Washington University Road Test administered by driving instructors from Independent Drivers, LLC
Edwards, 2010	1,248 participants aged 55 years and older (1,099 active drivers)	Maryland Older Drivers Project	Cohort study	Maryland	1998–2008	Driving cessation (time to cessation in months over 10-year period)	Self-reported driving cessation on the Mobility Questionnaire
Langford, 2013	1222 participants aged 70 years and older who were active drivers	Candrive II/Ozcandrive cohort	Cohort study (baseline data analyzed cross-sectionally)	British Columbia, Manitoba, Ontario and Quebec, Canada; Queensland, Australia; Wellington, New Zealand	2009–2014	Driving exposure (low mileage vs. high mileage drivers)	Self-reported annual driving distance
Marottoli, 1994	278 participants aged 72 years and older who were current drivers	Project Safety cohort	Cohort study	New Haven, Connecticut	1990–1991	Driving crashes and citations (crashes, moving violations, and being stopped by police in a 1-year period)	Participant responses at the 1-year follow-up interview
Stav, 2008	120 participants aged 65 and older who were current drivers	Participants recruited through physician referral and research at University of Florida’s National Older Driver Research and Training Center	Cross-sectional study	North Central Florida	Not specified	Driving ability (Global Rating Score assigned based on driving performance during a road test)	Global Rating Score assigned by a driving rehabilitation specialist
Woolnough, 2013	1230 participants aged 70 and older who were active drivers	Candrive II/ Ozcandrive cohort	Cohort study	British Columbia, Manitoba, Ontario and Quebec, Canada; Queensland, Australia; Wellington, New Zealand	2009–2014	Driving crashes (at-fault or not-at-fault crashes in the past 2 years)	Data on crashes obtained from provincial/state jurisdictions using participant driver license numbers

*SPPB* short physical performance battery, *TUG* timed up-and-go test, *RPW* rapid pace walk

**Table 2:** Exposures, covariates and outcomes for driving studies using the SPPB, the TUG, or the RPW as a measure of physical functioning

Measure	First author, Year	Exposures, participants, and covariates assessed	Outcomes measured
SPPB	Davis, 2011^a^	SPPB scores***, age***, sex***, education**, home circumstances (living alone or with others), BMI category, walking and mobility aid use**, IMD*, amenities within 5-min walking category, number of cars in household***	Driving exposure (number of car trips as a driver per week)
SPPB	Gill, 2012^b^	low SPPB score*, intermediate SPPB score, age (75-79y*, 80-84y*, ≥85*), female sex*, living with others, chronic conditions, moderate visual impairment, severe visual impairment*, weight loss*, cognitive impairment*, low physical activity*, lower-extremity weakness, gross motor coordination (8.8–10.3 s*, 10.4–12.4 s*, ≥12.5 s*), peak expiratory flow; precipitants: hospitalization* and restricted activity*	Driving cessation (long-term disability in driving a car, indicated by not driving in the past 6 months)
SPPB	Sims, 2007^c^	SPPB scores**, age*, sex, race, education, rural residence, SRH*, visual acuity, MMSE scores, GDS scores, CCI scores	Driving cessation
TUG	Dawson, 2010^d^	TUG, age*, sex, education, days driven per week, miles driven per week, CFT-Copy*, CFT-Recall*, Blocks*, BVRT, TMT A, TMT B, AVLT, JLO, COWA, COGSTAT**, UFOV, CS, FVA, NVA*, SFM, FR balance, Pegs*	Driving ability (safety errors per drive)
TUG	Emerson, 2012^e^	TUG, age*^a^, male gender, education**^b^, miles per week***^b^, number of crashes in past year, number of times pulled over in past year, exposure reduction score, intentional avoidance score, GDS, FR balance, Pegs*^a^, NVA*^a^, FVA, CS*^a^, JLO*^a^, SFM, UFOV*^a^, Blocks, CFT-Copy, CFT-Recall, BVRT*^a^, AVLT-Recall, TMT A*^a^, TMT B*^c^, TMT (B-A)*^c^, COWA, COGSTAT**^a^, overall road safety errors year 1, serious road safety errors year 1*^bc^	Driving cessation, citations, and crashes (time to driving event over a length of follow-up ranging from 3 to 8 years)
RPW	Ball, 2006^f^	RPW, age*, female sex*, history of at-fault crash involvement, history of falling*, delayed recall, tap time, MVPT**, TMT A, TMT B*, UFOV subtest 2**	Driving crashes (at-fault motor vehicle collision involvement during follow-up period of between 4.18 and 5.13 years)
RPW	Carr, 2011^g^	RPW, age, male sex, African American race, driving experience in years, days driven per week, miles driven per day, ≥1 crashes in previous year, FVA, CS*, presence of any abnormal score on visual field test, cervical range of motion left, cervical range of motion right, 9-Hole Peg Test right, 9-Hole Peg Test left*, grip strength right, grip strength left, brake reaction*, Short Blessed Test**, SMT**, CDT***, TMT A***, TMT B***, eight-item informant interview to differentiate aging and dementia total***, Digit Span Forwards, Digit Span Backwards**, MVPT, UFOV*	Driving ability (passing or failing the Washington University Road Test)
RPW	Classen, 2013^h^	RPW*, age, sex*, education, medication, MMSE, UFOV**, days of driving/week**, avoiding rush hour, avoiding the interstate*, avoiding rain, avoiding night driving, avoiding left turns, avoiding other	Driving ability (passing or failing the on-road driving test)
RPW	Edwards, 2010^i^	RPW, age*, days driven per week, MVPT, TMT B, UFOV*	Driving cessation (time to cessation in months over 10-year period)
RPW	Langford, 2013^j^	RPW*, gender*, age*, crash involvement in the last year, one leg stance (left leg), one leg stance (right leg), Ruler Drop*, Snellen visual acuity*, MMSE, Montreal cognitive assessment, MVPT*, TMT A*, TMT B*, Digit Span Forwards, Digit Span Backwards, months in reverse order*, self-rated abilities (see road signs at distance*, see road signs at distance at night*, see road lines at night*, see objects on road at night with glare or on wet roads*, quickly find street or exit in unfamiliar area and heavy traffic*, get in and out of car*), comfort in daytime driving situations (in light rain*, in heavy rain*, parking in tight spots*, in unexpected storm*, seeing street or exit signs with little warnings*, surrounded by multiple transport trucks*, tailgated by other drivers, passed by other drivers in non-passing lane, other drivers do not signal or seem distracted*)	Driving exposure (low mileage drivers [<5,001 km/yr] vs. high mileage drivers [≥15,000 km/yr])
RPW	Marottoli, 1994^k^	RPW, impaired design copying*, number of blocks walked*, number of foot abnormalities*, driving frequency, housing type	Driving crashes and citations (crashes, moving violations, and being stopped by police in a 1-year period)
RPW	Stav, 2008^l^	RPW***, MMSE***, UFOV***, TMT B***, Letter cancellation, Digit Span Forwards*, digit symbol substitution task, delayed recall, visual fields, acuity, MVPT spatial orientation subtask*, MVPT visual closure task, depth perception**, CS A***, CS B***, CS C***, CS D***, CS E***, Rules of the Road Test*, Road Sign Test***, right grip strength**, left grip strength*, trunk/neckrotation to left*, trunk/neck rotation to right**,	Driving ability (Global Rating Score assigned based on driving performance during a road test)
RPW	Woolnough, 2013^m^	RPW, Snellen visual acuity, visual field by confrontation, TMT B, CDT, neck rotation, shoulder and elbow flexion, finger curl, ankle plantar flexion, ankle dorsiflexion, shoulder adduction and abduction, wrist flexion and extension, hand-grip strength, hip flexion and extension, ankle dorsiflexion and plantar flexion	Driving crashes (at-fault or not-at-fault crashes in the past 2 years)

*SPPB* short physical performance battery, *IMD* index of multiple deprivation, *SRH* self-rated health, *MMSE* mini-mental state examination, *GDS* geriatric depression scale, *CCI* Charlson comorbidity index, *TUG* timed up-and-go test,*CFT-Copy* complex figure test-copy, *CFT-Recall* complex figure test-recall, *Blocks* WAIS-III block design, *BVRT* benton visual retention test, *TMT* trail making test, *AVLT* Rey auditory verbal learning test, *JLO* judgment of line orientation, *COWA* Controlled Oral Word Association, *COGSTAT* composite measure of cognitive function; *UFOV* useful field of view, *CS* contrast sensitivity, *FVA* far visual acuity, *NVA* near visual acuity, *SFM* structure from motion, *FR* functional reach, *Pegs* grooved pegboard test, *RPW* rapid pace walk, *MVPT* motor free visual perception test, *SMT* snellgrove maze test, *CDT* clock drawing test, *ADReS* assessment of driving related skills

^a^T-test and ANOVA analyses

^b^Cox proportional hazards regression reporting hazard ratios; reference values: SPPB score (high), age (70-74y), visual impairment (none or mild), gross motor coordination (≤8.7 s)

^c^Multivariable logistic regression analysis reporting adjusted odds ratios

^d^Multiple linear regression analysis of estimated changes in total driving safety errors for a 1-standard deviation increase in cognitive, visual, and motor predictors, controlling for age, education, and sex

^e^Cox proportional hazards regression reporting hazard ratios for a 1 standard deviation increase in visual, motor, and cognitive predictors, controlling for age, gender, education, and baseline mileage driven per week; 3 regression models for the 3 driving outcomes with significance indicated by ^a^driving cessation ^b^citations ^c^crashes

^f^Chi-squared test analyses for association between at-fault motor vehicle collisions and demographics and selected screening tests, all covariates adjusted for annual miles driven

^g^Correlations of demographic, noncognitive, and selected psychometric tests with failure on the road test

^h^Logistic regression reporting adjusted odds ratios

^i^Cox proportional hazards regression final model for time to driving cessation

^j^Bivariate comparisons between low mileage and high mileage drivers on demographics, physical/sensory performance, cognitive performance, and comfort with aspects of daytime driving

^k^Binomial relative risk modeling adjusted for driving frequency and housing type

^l^Correlations of independent variables with the Global Rating Score

^m^Fisher’s exact test, Pearson’s chi-squared test, and independent samples *t*-test analyses comparing those who were and were not involved in a collision on ADReS sub-tests

*p < .05, **p < .01, ***p < .001

### Study quality

Study quality was assessed via the Newcastle-Ottawa Quality Assessment Scale. Two authors independently scored the studies (LD and JC) and compared their quality ratings. The raters had nearly identical tables, with only two minor disagreements, which were resolved (LD yielded on one, JC on the other). If there were major discrepancies, then the plan was for the first author (TM) to intervene. Six of the seven cohort studies were deemed to be high quality, with an average assessment score of 8.1 out of 9 (range 7–9). The six cross-sectional studies varied in quality, with an average score of 7.3 out of 10 (range 6–8) (Table 3).

**Table 3:** Quality ratings for 7 cohort studies and 6 cross-sectional studies included on the basis of Newcastle-Ottawa quality assessment scale

Cohort studies									
	Representativeness of exposed cohort	Selections of non-exposed cohort	Assessment of exposure	Absence of outcome at start of study	Comparability	Assessment of outcome	Follow-up period (≥6 months)	Adequacy of follow-up	Total score out of 9 points
Ball et al., 2006	1	1	1	1	1	1	1	1	9 (high)
Edwards et al., 2010	1	1	1	1	2	0	1	1	8 (high)
Emerson et al., 2012	1	1	1	1	2	1	1	1	9 (high)
Gill et al., 2012	1	1	1	1	2	0	1	1	8 (high)
Marottoli et al., 1994	1	1	1	1	2	0	1	1	8 (high)
Sims et al., 2007	1	1	1	1	2	0	1	1	8 (high)
Woolnough et al., 2013	1	1	1	1	0	1	1	1	7 (medium)
Cross-sectional studies									
	Representativeness of sample	Sample size	Non-respondents	Ascertainment of primary measurement	Comparability	Ascertainment of the outcome	Statistical test		Total score out of 10 points
Carr et al., 2011	1	0	0	2	0	2	1		6 (medium)
Classen et al., 2013	1	0	0	2	2	2	1		8 (high)
Davis et al., 2011	1	0	0	2	2	2	1		8 (high)
Dawson et al., 2010	1	0	0	2	2	2	1		8 (high)
Langford et al., 2013^a^	1	1	0	2	0	1	1		6 (medium)
Stav et al., 2008	1	0	0	2	2	2	1		8 (high)

^a^Langford et al., 2013 used baseline data from a prospective cohort study and analyzed the data obtained in a cross-sectional manner

### Summary of findings

The driving outcomes that were obtained in conjunction with at least one physical functioning measure included: driving exposure, cessation, crashes, citations, and ability. Below is a description of results by each of the three included physical performance measures.

#### Short physical performance battery

A cohort study of UK participants aged 70 years and older was analyzed as across-sectional study focusing on driving exposure, measured as the number of car trips that were made each week by an individual as a driver (Davis et al. 2011). Lower SPPB scores were associated with reduced driving exposure [5.1 trips for older adults with high SPPB scores (10–12), 2.5 for those with intermediate SPPB scores (7–9), and 1.0 for those with low SPPB scores (<7) (ANOVA, *p* < .001 and t-tests not reported)]. Two US cohort studies focused on driving cessation as an outcome (Gill et al. 2012; Sims et al. 2007) in older adults 70 and 65 years, respectively. However, one of these specifically termed the outcome “long-term disability in driving a car,” which was indicated by not driving in the past six months (Gill et al. 2012). Specifically, Gill et al. (2012) found that low SPPB scores (4–6) relative to high SPPB scores (10–12) had an adjusted hazard ratio (HR) of 2.20 (95% CI 1.32–3.68) whereas intermediate SPPB scores (7–9) relative to high SPPB scores (10–12) had an adjusted HR for increased driving disability rate of 1.35 (95% CI 0.81–2.26). Sims et al. (2007) reported for every one-point decline in SPPB scores there was an 16% increased odds of driving cessation (adjusted OR 1.16, 95% CI 1.05–1.28). In summary,both studies concluded that lower SPPB scores were associated significantly with increased driving cessation or driving disability, respectively.

#### Timed up-and-go test

There are two TUG studies included. In both, the researchers called the TUG test by the original name the “Get-Up and Go”test, but specified that “time to completion” was the measure used. One Iowa cohort of aged 65 and older which included three driving outcomes: driving cessation, driving citations, and crashes (Emerson et al. 2012). The researchers called the citations “moving violations” and included citations that occurred when the car was in motion. Older adult participants completed two trials of the TUG test which were averaged. The TUG test completion time was not associated with driving cessation (adjusted HR 1.29, 95% CI 0.88–1.90), crashes (adjusted HR 1.29, 95% CI 0.96–1.72), or citations (adjusted HR 1.01, 95% CI 0.66–1.53). Another Iowa cross-sectional study focused on driving ability as a final outcome (Dawson et al. 2010). Driving ability was based on the number of driving errors made on a 35-mile route, and was not associated with TUG performance (from an adjusted multiple linear regression model a one standard deviation (SD) increase in TUG completion time resulted in .46 less safety errors, Standard Error (SE) = 1.27, *p* = .72).

#### Rapid pace walk

Three cohort studies included driving crashes as an outcome, with primarily null results (Ball et al. 2006; Marottoli et al. 1994; Woolnough et al. 2013). The US Project Safety cohort of aged 72 and older was the first to introduce the RPW as a physical functioning measure (Marottoli et al. 1994). The driving outcome in this study was a composite measure of driving crashes (fault not determined) and driving citations, including the self-report of being involved in a crash, receiving a moving violation, or being stopped by the police in the past year (Marottoli et al. 1994). Poorer RPW scores (completing the task in > 7 s compared to completing it in ≤ 7 s) were associated with a higher risk for the composite driving outcome in unadjusted (RR 2.0, 95% CI 1.0–3.8) analyses but not in an adjusted binomial risk model. The other two studies, one using the Maryland Older Drivers Project which included a cohort 55 years and older and the Candrive II/Ozcandrive which included a cohort of 70 years and older found no relationship between RPW scores and crashes (Ball et al. 2006; Woolnough et al. 2013).

Driving ability measured in various ways was a primary outcome in three cross-sectional studies (Carr et al. 2011; Classen et al. 2013; Stav et al. 2008). In one study of aged 65 and older Floridians, the RPW showed the strongest correlation with the Global Rating Score (*r* = −.454, *p* < .001) which was the driving ability measure assigned during a road test (Stav et al. 2008). The best predictor of poor driving ability was worse performance on the RPW. Another study of aged 65 and older Floridians measured driving ability with a comprehensive driving evaluation, with the outcome consisting of passing or failing the test (Classen et al. 2013). Poor RPW performance was associated (adjusted OR 1.45, 95% CI 1.05–2.00) with failing the driving test. The third study assessed driving ability in 52 year olds and older with dementia (mean of 5.3 on the Eight-item Informant Interview to Differentiate Aging and Dementia or the AD-8) who were also current drivers by whether or not an individual passed or failed a driving test, but did not find an association between RPW performance and driving ability [averaged score of 7.5 versus 8.3 s on the RPW for passing or failing the test] (Carr et al. 2011).

Langford et al. (2013) conducted a cross-sectional analysis from the Candrive II/Ozcandrive prospective cohort study for 1,222 participants including driving exposure as an outcome (measured as the participants’ self-reported annual driving distances from the previous year). The researchers did find a relationship between driving exposure and performance on the RPW, with low mileage drivers being more likely to be low RPW performers (RR = 1.30, 95% CI 1.08–1.55) and high mileage drivers being more likely to be high RPW performers (RR = 1.43, 95% CI 1.09–1.88). One prospective cohort study, the Maryland Older Drivers Project, included driving cessation as an outcome measure and found that RPW performance was not associated with driving cessation in the final Cox model (HR = 1.33, 95% CI 0.95–1.87), although poorer RPW performance was associated with an increased rate of driving cessation in an earlier physical performance Cox model (HR = 1.91, 95% CI 1.37–2.65) (Edwards et al. 2010).

## Discussion

This systematic review finds that lower scores on the SPPB are associated with increased driving cessation and reduced driving exposure, poorer performance on the RPW is associated with poorer driving ability in some studies and with reduced driving exposure in one study but is not convincingly associated with increased driving crashes, citations, or cessation, and poorer TUG test scores are not associated with any included driving outcomes. These limitations of the RPW and the TUG test can guide the use of these measures with specifically appropriate driving outcomes. The ability for the SPPB to be utilized successfully across multiple driving outcomes indicates that the SPPB is a promising measure and worthy of further study in driving research that may include additional driving outcomes.

Lower SPPB scores were consistently associated with reduced driving exposure and increased driving cessation (Davis et al. 2011; Gill et al. 2012; Sims et al. 2007). Physical performance is a modifiable risk factor and increases in SPPB scores can be accomplished through fitness interventions. A gain of only one point on this 12-point scale can be considered a substantial change (Kwon et al. 2009; Perera et al. 2014; Perera et al. 2006). A gain of one point could be accomplished by making progress on just one of the three included tasks (standing balance, walking speed, or ability to rise from a chair).

We posit that the SPPB could be the best measure of physical performance with driving exposure or driving cessation as the outcomes. More importantly, if we focus on upstream ways to keep older adults driving, then maintaining physical performance by physical activity and exercise may be our best buy. Although not the focus of this review, we know that physical activity and exercise can improve cognitive function across the lifespan and that both cognitive and physical function need to be maintained at a certain level to keep driving safely (Agency for Healthcare Research and Quality 2012; Carvalho et al. 2014; Colcombe et al. 2006; Kramer and Erickson 2007; Miller et al. 2016). Longstanding research supports physical functioning improvement (i.e., gait velocity and muscle strength) with exercise training at any age, indicating that physical functioning, as a modifiable risk factor, could be a promising focus for future interventions to assist older adults in maintaining safe driving (Fiatarone et al. 1994; Nelson et al. 1994, Pahor et al. 2014). Various exercise and physical activity interventions can be implemented with older adults to keep them driving longer, with SPPB used as a measure of change. As previously mentioned, limited research is exploring what types of exercise interventions could also be specifically beneficial for maintaining or improving which kinds of driving ability (Marmeleira et al. 2011; Marmeleira et al. 2009; Marottoli et al. 2007).

Regarding safety, one TUG test study and three RPW studies included crashes as an outcome (although the Marottoli et al. RPW study actually included a composite measure of crashes and citations) (Ball et al. 2006; Emerson et al. 2012; Marottoli et al. 1994; Woolnough et al. 2013). Poorer performance on the physical performance measures (TUG and RPW) was not associated with increased crashes in any of these studies. It is worth noting that studies with small sample sizes may have trouble assessing crashes, which are not a frequent outcome. However, only the TUG test study (Emerson et al. 2012) had a relatively small sample size of 98 participants with crash data. The other crash studies included here reported higher sample sizes: 1,910 (Ball et al. 2006), 278 (Marottoli et al. 1994), and 1,230 (Woolnough et al. 2013). Another important crash factor that should be considered is the difference between at-fault and not at-fault crashes. The only study that included strictly at-fault crashes was Ball et al. (2006). The other studies looked at overall crashes that may have been at-fault or not at-fault, which may account, in part, for the lack of association observed.

Two of the RPW driving ability studies found that poorer RPW performance was associated with poorer driving ability, and one study did not (Carr et al. 2011; Classen et al. 2013; Stav et al. 2008). The one TUG driving ability study did not find poorer TUG performance to be associated with poorer driving ability (Dawson et al. 2010). One RPW study included driving cessation as an outcome and did not find a link between poorer RPW scores and increased driving cessation in their final analyses (Edwards et al. 2010). Another RPW study included a driving exposure outcome and determined that low mileage drivers are likely to have worse completion times on the RPW, whereas high mileage drivers are likely to have better completion times on the RPW (Langford et al. 2013). Like the SPPB, improvement on the RPW can be accomplished through exercise interventions.

This review did not find any association between the TUG test and driving outcomes (increased driving cessation, crashes, or citations, or decreased driving ability), but this could be due to the limited number of studies (two) and their small sample sizes (100 and 111 participants) (Dawson et al. 2010; Emerson et al. 2012). It is worth considering if there are specific differences in the administration of the TUG test compared to the RPW and the SPPB that could account for these differences in our findings. Compared to the other two, the TUG test has less standardization across protocols. Differences between published guidelines of the TUG test, include: using a cone on the floor versus a line, walking as quickly as possible versus walking at a normal pace, recording the fastest of two trials versus recording only one trial, and starting from an arm chair versus a folding chair (American College of Rheumatology 2015; Centers for Disease Control and Prevention 2015; Rikli and Jones 2001). The distance travelled varies in protocols from “8 ft” (2.44 m) to “3 m” (9.84 ft) to “3 m or 10 ft” (9.84 ft or 3.05 m) (American College of Rheumatology 2015; Centers for Disease Control and Prevention 2015; Rikli and Jones 2001).

Study types included cross-sectional and cohort designs. Including studies with a cross-sectional design has the major limitation of not being able to establish temporality between the physical performance measures and the driving outcomes. The scarcity of studies with longitudinal designs was the rationale for including this design; future reviews should consider using only longitudinal designs. The heterogeneity present from a 50 year old to an 80 year old is a limitation. We selected adults 50 years and older because of current CDC programs such as the CDC’s Healthy Aging Program. Stratification by age is warranted as the literature expands on this topic to assess the effects of being “young old” versus “old old.” The majority of the included studies (12/13) included at least 100 participants, and the one study that did not included 99 (Carr et al. 2011). That study also included only older adults who had been diagnosed with dementia, which does not represent the average older adult population. Since both life expectancy and the prevalence of dementia will be increasing, it will be important to include targeted medical populations such as those diagnosed with dementia in future reviews to see which physical performance measure can be used successfully in these heterogeneous populations to measure specific driving outcomes (Satizabal et al. 2016). Many of the driving outcomes examined in this review are rare (e.g. crashes) in the general older adult population and, due to this low prevalence, is may be difficult for these physical performance tests to identify risks. If used among older adult populations who may have strength and balance problems (such as among individuals with arthritis, Parkinson’s disease, etc.), researchers may be more likely to observe a direct association between some of the physical performance measures and driving outcomes.

Future driving studies should pay careful attention to the standardization of measures. One of the three SPPB studies made modifications by changing the number of chair stands and using a timed rapid gait measure instead of a timed usual gait measure (Gill et al. 2012). While the authors recognize that modifying existing measures can sometimes be advantageous for specific studies, we advocate here that when there is no risk of compromising the study’s aims, researchers should consider using the SPPB unmodified so that there can be more standardization across the field, allowing for better comparability between studies.

Both TUG test studies called the TUG by the original name: the “Get-up and Go” test and the test was only identified via a reference to Podsiadlo and Richardson or to a mention of the timed nature of the measure (Dawson et al. 2010; Emerson et al. 2012; Podsiadlo and Richardson, 1991). Additionally, in one study the average score of two trials was recorded (Emerson et al. 2012). The reviewers recommend further standardizing the TUG test in practice.

The SPPB, TUG test, and RPW are physical functioning measures; performance on these measures may be impacted by other aspects of health and functioning. Again, a recent review stresses the established benefits of physical function on cognitive function in older adults (Miller et al. 2016; Colcombe et al. 2006; Kramer and Erickson 2007). Research has found that performance on the TUG test is associated with poorer vision and cognitive impairment, as another example (Aartolahti et al. 2013; Ayan et al. 2013; Eggermont et al. 2010). To proceed from that point, this review was concerned only with the association between driving outcomes and complete physical performance tests. Future inquiry into this topic may wish to examine associations between driving outcomes and the individual components of these tests (standing balance, usual and rapid walking speed, and chair stands) separately.

## Conclusions

The SPPB was associated with two of the driving outcomes in this review, but the research is still sparse and the driving outcomes limited. More longitudinal studies are needed to confirm the potential association of SPPB scores and other mobility measures with driving outcomes. Despite its respected position in older adult falls research, the TUG test did not prove to be a useful measure in the driving research here, which included a wide range of driving outcomes, although studies were limited. Perhaps more standardized protocols to train assessors and implement the TUG test across studies would improve the precision to measure change in driving outcomes. The RPW appeared to be a useful measure for studies that include driving ability or exposure as an outcome, but it may not be useful in predicting crashes or citations.

The importance of continued motor vehicle driving for the mental, physical, and social well-being of older adults, as well as the importance of preventing crashes, is established. Longstanding research in aging populations supports the notion that physical functioning can improve with exercise interventions. This review also supports future interventions that target physical performance improvements in order to maintain safe and continued driving for older adults.

## Abbreviations

MeSH: Medical Subject Heading
PRISMA: Preferred Reporting Items for Systematic Reviews and Meta-Analyses
RPW: Rapid pace walk
SPPB: Short physical performance battery
TUG: Timed up-and-go test
